# Electrodermal reactivity in an aversive countdown task: Concurrent and prospective relations with triarchic psychopathy traits and antisocial behavior outcomes in a child-aged sample

**DOI:** 10.3389/fpsyt.2025.1680358

**Published:** 2025-12-03

**Authors:** Bridget M. Bertoldi, Christopher J. Patrick, James R. Yancey, Rachel LaManna, Catherine Tuvblad, Sofi Oskarsson, Laura A. Baker, Adrian Raine, Don Fowles

**Affiliations:** 1 Worcester Recovery Center and Hospital, Worcester, MA, United States; 2 Department of Psychology, Florida State University, Tallahassee, FL, United States; 3 VA Salt Lake City Health Care System, Salt Lake City, UT, United States; 4 School of Behavioural, Social and Legal Sciences, Orebro universitet, Örebro, Sweden; 5 Department of Psychology, University of Southern California, Los, Angeles, CA, United States; 6 Department of Psychology, University of Pennsylvania, Philadelphia, PA, United States; 7 Department of Psychology, The University of Iowa College of Liberal Arts and Sciences, Iowa City, IA, United States

**Keywords:** psychopathy, skin conductance, triarchic traits, antisocial behavior, longitudinal

## Abstract

**Introduction:**

Considerable evidence exists for reduced electrodermal reactivity to aversive cues/events in high-psychopathic individuals, but most research of this kind has employed adult samples and cross-sectional designs. The current study examined skin conductance (SC) activation during anticipation of and in response to a noise stressor in a sample of 9–10 year old children in relation to constituent traits of psychopathy described by the triarchic model (i.e., boldness, disinhibition, meanness), both concurrently and at a five-year follow-up, and in relation to antisocial behavior at the follow-up.

**Methods:**

Participants were 1,082 children from the greater Los Angeles area who underwent successive waves of testing in a longitudinal twin project. Current study analyses focused on (a) SC from the noise stressor task and parental ratings of children’s triarchic traits collected at wave 1 of the project (W1; ages 9-10), and (b) parent- and child-ratings of the triarchic traits along with child-reported engagement in aggressive and nonaggressive antisocial behavior (ASB) collected at wave 3 (W3; ages 14-15).

**Results:**

Reduced SC reactivity in the noise-stressor task at W1 was related to parent-rated boldness both concurrently (at W1) and prospectively (at W3), but not to parent-rated disinhibition or meanness at either wave. Interestingly, reduced SC reactivity at W1 showed associations with both boldness and meanness as rated by child participants themselves at W3. Additionally, reduced W1 SC reactivity was uniquely predictive of child-reported nonaggressive ASB at W3, whereas W1 parent-rated boldness was uniquely predictive of child-reported aggressive behavior at W3.

**Discussion:**

Our finding of an inverse relationship between SC reactivity and parent-rated boldness at W1 corroborates evidence from prior adult research and points to low fear (threat sensitivity) as the basis of deficient stress responding in psychopathy. The finding of contrasting predictive relations for W1 SC reactivity and W1 parent-rated boldness with ASB at W3 suggests differing roles for elements of threat sensitivity tapped by each (e.g., low anxiousness versus social dominance) in nonaggressive versus aggressive forms of ASB. Our findings highlight the nuances of dispositional fearlessness as construct of relevance to psychopathy and suggest important avenues for future research.

## Introduction

Psychopathy is a clinical condition marked by impulsive deviancy (Factor 2) symptoms, including antisocial behavior, in the presence of distinct affective and interpersonal (Factor 1) features. The affective-interpersonal features include a lack of emotional sensitivity and impaired social relatedness (1, 2). These deficits are observed not only behaviorally, but also physiologically, with psychopathic individuals exhibiting affective response deficits including *reduced* autonomic responsiveness – electrodermal reactivity, in particular – to threatening or aversive stimuli in laboratory studies. However, much of the existing research examining relations between psychopathic features and affective-physiological reactivity has focused on incarcerated offenders [for reviews, see: (3–5)] or adult non-offenders [e.g., (6, 7)]. More work is needed with younger-aged community samples assessed for psychopathic characteristics. The current study was undertaken to address this need.

## Psychopathy and electrodermal reactivity to aversive stimuli: Adult studies

Psychophysiological studies of adult offenders diagnosed as psychopathic on the basis of global diagnostic ratings [see (5)] or scores on Hare’s (8) Psychopathy Checklist-Revised [PCL-R; see (4, 9)] have reported lowered autonomic reactivity during anticipation of aversive stimuli (electric shock, loud noise) in terms of reduced skin conductance response (SCR), a widely used measure of sympathetic nervous system activation (10). Additionally, in some studies, adult criminal offenders scoring high in psychopathy have been found to exhibit blunted SCR to actual presentations of aversive stimuli [e.g., (11–13)]. These findings suggest that psychopathy as manifested in offender samples is marked by reduced physiological reactivity to cues signaling aversive events, and perhaps to aversive events themselves (4, 9).

Research with non-offenders has also provided evidence for decreased SC reactivity to aversive stimuli in relation to psychopathic propensities as indexed by the Psychopathic Personality Inventory (PPI; 14, 15). Benning et al. (16), for example, found that adolescent males from the community scoring high on the PPI’s fearless dominance factor (PPI-1), a trait dimension related to the interpersonal-affective features of PCL-R psychopathy (6, 17), exhibited blunted SCR to aversive but not pleasant or neutral picture stimuli. Paralleling this, Dindo & Fowles (7) reported reduced SC during anticipation of an aversive noise-blast stimulus in male undergraduates scoring high on the PPI’s fearless dominance factor; by contrast, those scoring high on the self-centered impulsivity factor of the PPI – indicative of impulsive-deviancy features of psychopathy – exhibited *increased* SCR during a social stressor task (i.e., delivery of a speech). Selective associations of diminished SCR with the interpersonal-affective features of psychopathy have also been reported in studies with non-offenders that have used other aversive cueing paradigms (e.g., differential fear conditioning; 18).

A key question arising from findings of these studies of offenders and nonoffenders is what underlying biological characteristic of individuals scoring high on interpersonal-affective features of psychopathy accounts for their reduced aversive-SC reactivity. A conceptual model that has shown promise for addressing this question is the triarchic model of psychopathy (19), which conceptualizes symptom facets of this condition in biobehavioral-trait terms. Specifically, the model posits that biobehavioral traits of boldness and meanness (or callous unemotionality; 20) underlie the affective-interpersonal symptoms of psychopathy, whereas the trait of disinhibition (externalizing proneness; 21) underlies the impulsive-deviancy features. Boldness, which correlates very strongly with the fearless dominance factor of the PPI (22) and uniquely with the PCL-R’s interpersonal features (23), is theorized to reflect low sensitivity of the brain’s defensive (threat) mobilization system (19). Consistent with this, boldness scale scores correlate highly with self-report measures of fearlessness, threat sensitivity, and tolerance for uncertainty/danger (6, 24). The second triarchic model trait, meanness, encompasses deficient empathy, exploitativeness, and lack of close attachments, and is theorized to entail a specific weakness in affiliative capacity (25, 26). It correlates uniquely with the PPI’s Coldheartedness subscale and relates to both the affective and antisocial symptom facets of the PCL-R (23). The third triarchic trait – disinhibition – reflects weak restraint, irresponsibility, and boredom proneness, and correlates most strongly with the PPI’s impulsive-deviancy facet and the PCL-R’s impulsive facet, and secondarily with the PCL-R’s antisocial facet (22, 23). Deficits in behavioral and affective control associated with this trait characteristic are theorized to reflect dysfunction in frontal-regulatory circuits of the brain (27, 28).

Since the emergence of the triarchic model, a growing body of research has provided evidence for blunted autonomic reactivity to aversive stimuli specifically in relation to the triarchic trait of boldness. An initial demonstration of this was reported by Benning et al. (16), who as noted earlier reported reduced SCR to aversive picture stimuli in relation to Factor 1 of the PPI, which reflects boldness (22). Another study by Kyranides et al. (29) found that boldness as assessed by the Triarchic Psychopathy Measure (TriPM; 30) was associated with reduced heart rate reactivity (HRR) to aversive picture stimuli – an effect not evident for either meanness or disinhibition. Other work by Yancey et al. (31, 32) has shown that individuals high in TriPM-assessed boldness are resistant to the impairing effects of imminent threat on cognitive task performance. Taken together, these findings provide evidence for boldness relating to reduced defense-system response in adult-aged participants.

In sum, findings from studies of adult offenders and nonoffenders provide consistent evidence of decreased skin conductance reactivity to nonsocial stressors (cues signaling the possibility of shock or loud noise; aversive visual images) in relation to Factor 1 psychopathy features, with one nonoffender study (7) reporting increased SCR to a social stressor (speech performance) in relation to Factor 2 features.

## Psychopathy and electrodermal reactivity to aversive stimuli: Findings for younger-aged samples

Counterpart research on psychopathy and electrodermal reactivity to aversive stimuli in child and adolescent samples is more limited. Available studies of this kind, which have employed Lynam’s (33) Child Psychopathy Scale (CPS) to index psychopathic propensities, have only partly corroborated findings from adult studies. One such study, by Fung et al. (34), examined relations between CPS-assessed psychopathy and autonomic reactivity to threat cues in a procedure termed the ‘countdown’ task (11), in which participants view a sequential series of numbers signaling the upcoming occurrence of a stressor at some point in the series. These authors found that male adolescents with high total CPS scores exhibited reduced SCR both in anticipation of and in response to loud noise blasts. However, Fung et al. did not examine relations of SCR with subdimensions of the CPS corresponding to Factors 1 and 2 of psychopathy. Another study of male youth by Wang et al. (35), also employing this countdown-to-noise task, did examine reactivity in relation to two factors of the CPS labeled Manipulative/Deceitful (M/D) and Callous/Disinhibited (C/D), identified via exploratory factor analysis in prior work by Bezdjian et al. (36). Wang et al. reported fewer non-specific skin conductance responses (NS-SCRs) within the countdown task in boys high on the M/D factor of the CPS, encompassing features ostensibly related to boldness (e.g., glibness, persuasiveness, conning). By contrast, these authors found that higher scores on the CPS’s C/D factor, reflecting callousness and shallow affect along with boredom proneness and impulsivity/disconstraint, were related to increased HR acceleration during stressor anticipation. One other study by Isen and colleagues (37) that examined electrodermal reactivity to non-cued acoustic stimuli in relation to these two CPS factors found reduced SCR among boys scoring high on the M/D factor, with no association evident for the C/D factor – a result that ostensibly aligns with adult research findings. However, the acoustic stimuli in this study were non-aversive (i.e., tones, naturalistic sounds) rather than aversive in nature.

There is a growing body of research on psychological and clinical correlates of the triarchic model traits in children and adolescents [see, e.g., (38–40)]. These studies have largely corroborated, and importantly extended, research with adults demonstrating theory-consistent relations of the triarchic traits with various self-report and interview-based measures of personality, behavioral propensities, and psychopathology. However, minimal research exists on the physiological correlates of the triarchic traits in youth. One study by Bertoldi et al. (41) reported evidence of a relationship between low resting heart rate in childhood (age 9-10) and later engagement in antisocial behavior (at age 19-20) that was jointly mediated by the triarchic traits of boldness and disinhibition. However, no study has yet tested for relations of the triarchic traits with autonomic reactivity to emotionally evocative stimuli.

To summarize, published research examining autonomic reactivity in anticipatory threat (countdown) tasks in younger-aged samples assessed for psychopathy, while informative, has been limited. No research has yet examined aversive stimulus reactivity in relation to the traits of the triarchic model in children or adolescents. The triarchic model is advantageous because it conceptualizes distinct trait facets of psychopathy in biobehavioral terms, and there is a growing literature on each triarchic trait’s physiological correlates (for a recent review, see 42). Systematic research is needed on autonomic stress reactivity in relation to the triarchic model traits in developmentally sensitive samples to build upon the existing literature.

## The current study

The current study employed a longitudinal study sample and previously validated scale measures of the triarchic model traits (38) to test for associations of electrodermal (SC) reactivity from an aversive (loud noise) countdown task with these traits—both concurrently and prospectively. Participants were tested initially at ages 9-10, and at four successive follow-up intervals of 2–3 years each (for details, see 43). Study measures from the first assessment wave (W1; ages 9-10) consisted of SCR data from the countdown task for child participants and triarchic trait ratings collected from their parents; measures from the third assessment wave (W3; ages 14-15) consisted of triarchic trait data collected from participants as well as their parents, and criterion measures of antisocial behavior (ASB) collected from participants.^1^

Our major study hypotheses were for the triarchic trait of boldness. Specifically, we predicted that: (1) anticipatory SCR and (more tentatively) noise-evoked SCR from the W1 countdown task would correlate negatively with parent-rated boldness assessed concurrently (at W1), and with both parent- and participant-rated boldness assessed prospectively (at W3); (2) SCR from the W1 countdown task and parent-rated boldness at W1 would each predict participant ASB assessed prospectively, at W3. Our predictions for SCR and the other two triarchic traits, disinhibition and meanness, were tentative given the limited evidence for these traits relating to differential aversive-SC reactivity. For disinhibition, we hypothesized that: (3) in line with the above-mentioned finding for high impulsively deviant individuals reported by Dindo and Fowles (7), children rated high on this triarchic trait by parents at W1 might show enhanced SC reactivity in the W1 countdown task. For meanness, we hypothesized even more tentatively that: (4) parent-ratings for this trait at W1 would relate negatively to countdown-SCR, but at a lower magnitude than boldness. This fourth hypothesis was based on the triarchic model’s view that dispositional fearlessness is contributory but not central to meanness (19).

## Methods

### Participants

Study participants were child-aged twins and triplets from the University of Southern California (USC) Longitudinal Study of Risk Factors for Antisocial Behavior (RFAB) project (43). Data collection for the project occurred across five waves, with the first occurring during 2000–2004 when participants were 9 to 10 years of age, and subsequent waves occurring every 2 to 3 years thereafter, with the most recent (Wave 5) occurring at ages 19-20. The W1 base sample (*N* = 1673, 51.4% female; *M* age = 9.60 years at W1) was representative of the greater Los Angeles area in terms of race/ethnicity and socioeconomic status [for further details regarding sample characteristics, see (43); for details regarding recruitment and study inclusion criteria, see (44, 45)]. Skin conductance data were collected from 1152 (69%) of W1 participants, of whom 9 were excluded from analyses due to excessive artifact (i.e., large baseline skin conductance fluctuations).

Analyses were performed to test for concurrent relations of W1 countdown SC reactivity with W1 parental ratings of children’s triarchic traits, and prospective relations of each with parent- and child-rated traits and ASB scores from W3. Parent-rated traits from W1 were employed in analyses because: (i) these were expected to be more valid than child self-ratings at the youngest assessment age (9–10 years); (ii) as noted below, only partial item data were available for child-rated boldness at W1 (for further details, see 38); and (iii) use of W1 parent ratings avoided measurement-modality overlap with W3 child-rated triarchic traits and ASB scores. Given our use of these variables, the analysis sample comprised child participants with W1 countdown data for whom parent-rated triarchic trait scores were available at either W1 or W3, and/or for whom child participant-rated triarchic trait scores were available at W3. This resulted in a sample of 1,082 participants (51.8% female; *M* age = 9.60; 2% Native American, 4.5% Asian, 13.7% Black, 38% Hispanic, 26% White, and 17.7% Multiracial) for the analyses focusing on W1 parent ratings, and 681 participants (54% female; *M* age = 14.83; 5.3% Asian, 11.9% Black, 17.2% Mixed, 29% White, 36.6% Hispanic) for the analyses focusing on both W3 parent and child-participant ratings.

Informed consent was acquired from adult caregivers of all participants at W1 (ages 9-10) of the RFAB project. All study procedures were approved by the Institutional Review Board at USC.

### Skin conductance measurement

Skin conductance activity was recorded from bipolar leads on the distal phalanges of the index and middle fingers of participants’ non-dominant hand using silver-silver chloride electrodes 8 mm in diameter. A water-soluble gel was used, supported by an adhesive electrode collar (8 mm in diameter) that maintained full contact with the skin. Waterproof tape was subsequently wrapped around the finger and electrode for further security. Participants were instructed to keep this hand still during testing.

For the purpose of artifact checking, sec-by-sec SC values were first output and plotted for each subject, to allow for fine-grained visual inspection. Segments of the 233 s SC plot that contained extreme values – presumably due to movement or other recording irregularities – were flagged for exclusion, and participants with less than 1 min of useable SC data for the task period (*n* = 2) were omitted from analyses.

### Questionnaire measures

#### Risk Factors for Antisocial Behavior (RFAB) Triarchic scales

Since the development of the Triarchic Psychopathy Measure (30), multiple other self-report scale measures have been developed to assess the triarchic traits (see, e.g., 46–50). The RFAB-Triarchic (-Tri) scales were developed to index the trait constructs of the triarchic model within the RFAB sample, and the initial article on these scales (38) reported evidence of their validity in terms of strong convergent relations (*r*s = .6 –.7) with corresponding scales of the TriPM. Additional evidence of validity has been provided by longitudinal work reporting prospective relations for earlier-age scores on RFAB-Disinhibition, and to a lesser extent Meanness, with later-age attention deficit hyperactivity disorder (ADHD) symptomatology (51). Each RFAB-Tri scale includes items from the Child Psychopathy Scale (33) along with items from age-appropriate self-report versions of inventories from the Achenbach System of Empirically Based Assessment (ASEBA; 52, 53).^2^ The RFAB Boldness scale contains 10 items (six CPS items and four ASEBA items), but because only a parent-rating version of the ASEBA inventory exists for children aged 9-10, only partial item data for this and the other RFAB-Tri scales were available for child participants at W1; data for all 10 RFAB-Boldness scale items were available for parents at both W1 and W3, and for child participants at W3. For each scale, scores for constituent items were first z-transformed (to accommodate differing response formats) and then averaged to form a standard-score composite.

Internal consistency reliabilities (*α*) for the parent-rated RFAB Boldness scale were .62 at wave 1 and .60 at wave 3; W1 and W3 reliabilities for the parent-rated Disinhibition and Meanness scales were .76/.79 and .72/.74, respectively. Reliabilities for the child-rated Boldness, Disinhibition, and Meanness scales at W3 were .66, .72 and .69, respectively. Intercorrelations among scores for the parent-rated scales, at W1/W3, were: Boldness with Meanness, -.03/.20; Boldness with Disinhibition, -.09/.07; Meanness with Disinhibition, .44/.50. Corresponding intercorrelations among the child-rated scales, at W3, were: Boldness with Meanness, .04, Boldness with Disinhibition, -.02, and Meanness with Disinhibition, .43.

#### Youth Self-Report (YSR) questionnaire

At W3, data for 628 child participants were available for the 1991 version of the Youth Self-Report questionnaire, a 112-item ASEBA inventory that assesses various types of behavioral, emotional, and social competencies in children aged 11–17. Using data for this inventory, an aggressive ASB composite was created by summing scores for 20 YSR items pertaining to physically aggressive acts (*M* = 7.59, *SD* = 5.54; *α* = .85), and a nonaggressive ASB composite was created by summing scores for 13 YSR items pertaining to delinquent, rule-breaking deviancy (*M* = 3.15, *SD* = 2.74; *α* = .64).

### Countdown task (Wang, Baker, Gao, Raine, & Lozano, 2012)

The countdown task typically includes signaled trials only, wherein descending numbers appear prior to the delivery of aversive stimulation (e.g., loud noise or shock); previous work has utilized signaled trials in particular to assess both anticipatory fear and fear reactivity in psychopathic and non-psychopathic individuals (54–56). However, the countdown task utilized in the RFAB project included three signaled (countdown) trials along with two unsignaled trials on which no numbers preceded aversive stimulus delivery (34); the task as a whole lasted 233 s, with the signaled trials starting at 7 s, 94 s, and 188 s into the procedure, and the unsignaled trials starting at 48 s and 139 s.

Analyses focused on data for the three signaled trials, given their clearer connection to prior published work. For each of these trials, participants viewed numbers starting at 12 on a computer screen decrease by one each successive second until a loud noise (1-s burst of 105 dB white noise) was delivered when the number zero appeared. Anticipatory fear was quantified in terms of participants’ SC reactivity during the period of the numeric countdown, whereas defensive reactivity to the noise stressor was quantified as SCR to the occurrence of the noise blast itself. For anticipatory reactivity, the maximum SC value reached within the first 4 s of each signaled countdown trial was identified and subtracted from the mean for the initial second of the trial (i.e., 1-s period between onset of the number 12 and onset of the number 11). Noise-blast reactivity was computed for each trial by subtracting the maximum SC value occurring within the 6-s period following the onset of the noise blast from the mean for the 1-sec period immediately following the onset of the blast.

### Data analyses

We tested our study hypotheses for countdown-task SCR and its relations with the triarchic traits using mixed-general linear models (GLMs) implemented in SPSS version 23. Separate analyses were conducted for anticipatory SCR and noise-evoked SCR, with each model including a 3-level within-subjects Trial factor reflecting scores for one or the other SCR variable derived from each of the three signaled trials. Models examining relations of the triarchic traits with each SCR variable included the relevant trait score as a continuous between-subjects variable. Greenhouse-Geisser corrected statistical significance values are reported where necessary to account for violation of sphericity. To test for predictive relations of countdown-SCR and parent-rated triarchic traits at W1 with aggressive and nonaggressive forms of participant-rated ASB at W3, separate hierarchical regression analyses were conducted in which one or the other W1 SCR variable and one or another of the parent-rated traits were entered at steps 1 and 2, respectively, as predictors of W3 aggressive or nonaggressive ASB.

## Results

### Overall sample effects for wave 1 countdown task

**Figure 1:**
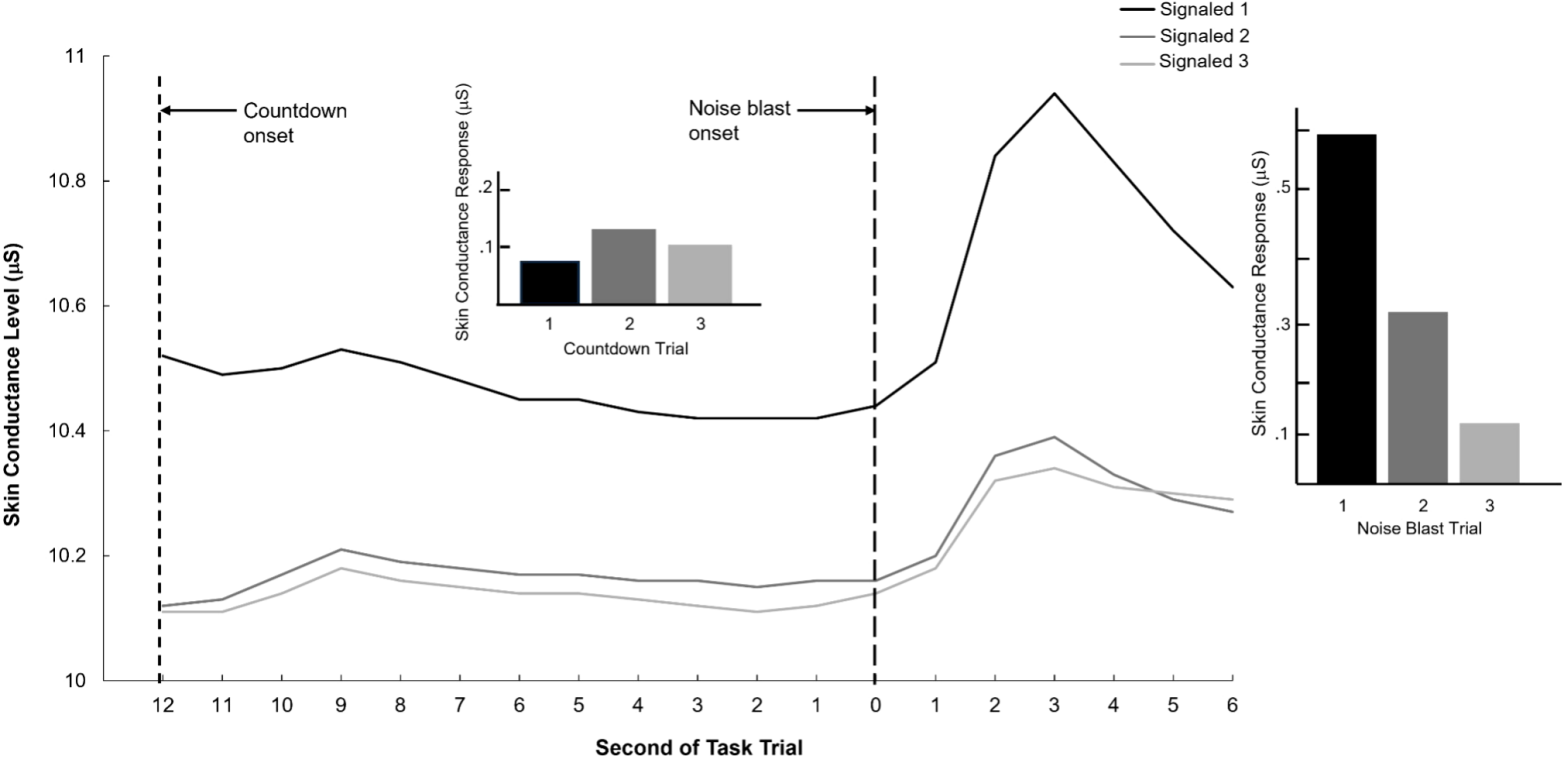
Line plot of mean raw skin conductance level (in microSiemens) for child participants as a whole (*N* = 1,082) for the three signaled trials of the Wave 1 countdown task, with left and right inset bar plots depicting, respectively, mean skin conductance reactivity (change from 1-s proximal baseline) for the numeric countdown and noise-blast presentation periods of each trial; the left and right vertical dashed lines denote the onsets of the 12-s countdown period and the 1-s noise blast presentation. The left bar plot shows, for each trial, the maximum increase in skin conductance level, relative to the first second of the countdown period (during which the number 12 appeared on-screen), over the initial 4 seconds of the countdown (through to the offset of the number 9). The right bar plot shows, for each trial, the maximum increase in skin conductance level, relative to the 1-s period of the noise-blast presentation, over the 6-s period following the onset of the blast.

In the one-way GLM examining anticipatory SC across the three signaled (i.e., countdown) trials, a significant main effect of trial was observed, *F*(1, 2) = 11.24, *p* < .001, *η*^2^ = .01. *Post-hoc* comparisons with a Bonferroni correction revealed that anticipatory SCR during the countdown period of the first trial was significantly lower compared to countdown periods for the second (*p* < .001) and third signaled trials (*p* = .01), which did not differ from one another (*p* = .11). Means and standard deviations for these variables are presented in the upper part of **Table 1**; **Figure 1** depicts average SC level across the two phases of the countdown procedure (anticipation, noise-blast delivery), by signaled trial (1-3).

**Table 1:** Descriptive statistics for skin conductance (μSiemens) during anticipation (Countdown) and in response to presentation of noise.

	*M*	*SD*	Range
Noise anticipation			
SC			
Sig 1	.09	.22	.00 – 2.61
Sig 2	.13	.29	.00 – 2.56
Sig 3	.11	.24	.00 – 2.01
Noise presentation			
SC			
Sig 1	.60	.63	.00 – 3.74
Sig 2	.31	.48	.00 – 4.12
Sig 3	.15	.29	.00 – 2.44

*N* = 1,082. Sig, signaled noise blast #; SC, skin conductance.

In the counterpart GLM for SCR to the noise blasts, a significant main effect of trial was observed, *F*(1, 2) = 380.06, *p* < .001, *η*^2^ = .26. Follow-up comparisons indicated that SC reactivity to the noise blast was greater on the first than either the second (*p* < .001) or the third signaled trial (*p* < .001), with noise-blast reactivity on the second signaled trial also significantly exceeding reactivity on the third (*p* < .001; see **Figure 1**). Means and standard deviations for these variables are presented in the lower part of **Table 1**.

### Concurrent associations: wave 1 countdown task reactivity with parent-rated triarchic traits

To evaluate whether autonomic reactivity on signaled trials of the W1 countdown task related to each triarchic trait assessed concurrently via parent ratings, we conducted two-way mixed model GLMs with each W1 trait included as a between-subjects factor along with signaled trial number (1-3) as a within-subjects factor. For anticipatory SC during countdown periods preceding the noise blast, the analysis for boldness yielded a significant main effect for this trait, *F*(1, 1,080) = 5.17, *p* = .02, with children exhibiting lower anticipatory SC prior to blast presentation across the three signaled trials scoring higher in parent-rated boldness at W1 (*r* = -.07). The corresponding main effect for W1 parent-rated boldness in the analysis for SCR to the noise blast itself was in the same direction, with children who exhibited less SCR to the noise blast across signaled trials tending to score higher in parent-rated boldness at W1 (*r =* -.06), but fell short of significance, *F*(1, 1,080) = 3.54, *p* = .06. No evidence of a Boldness x Trial interaction was found in either of these analyses (*p*s > .79). The counterpart GLMs for disinhibition and meanness yielded no main or interactive (i.e., trait by trial) relations for either with SC during anticipation of or in response to the signaled noise blasts, all *F*s < 1.45, *p*s > .23, *r*s for disinhibition/meanness with anticipatory/noise-blast SCRs < |.03|.

**Figure 2:**
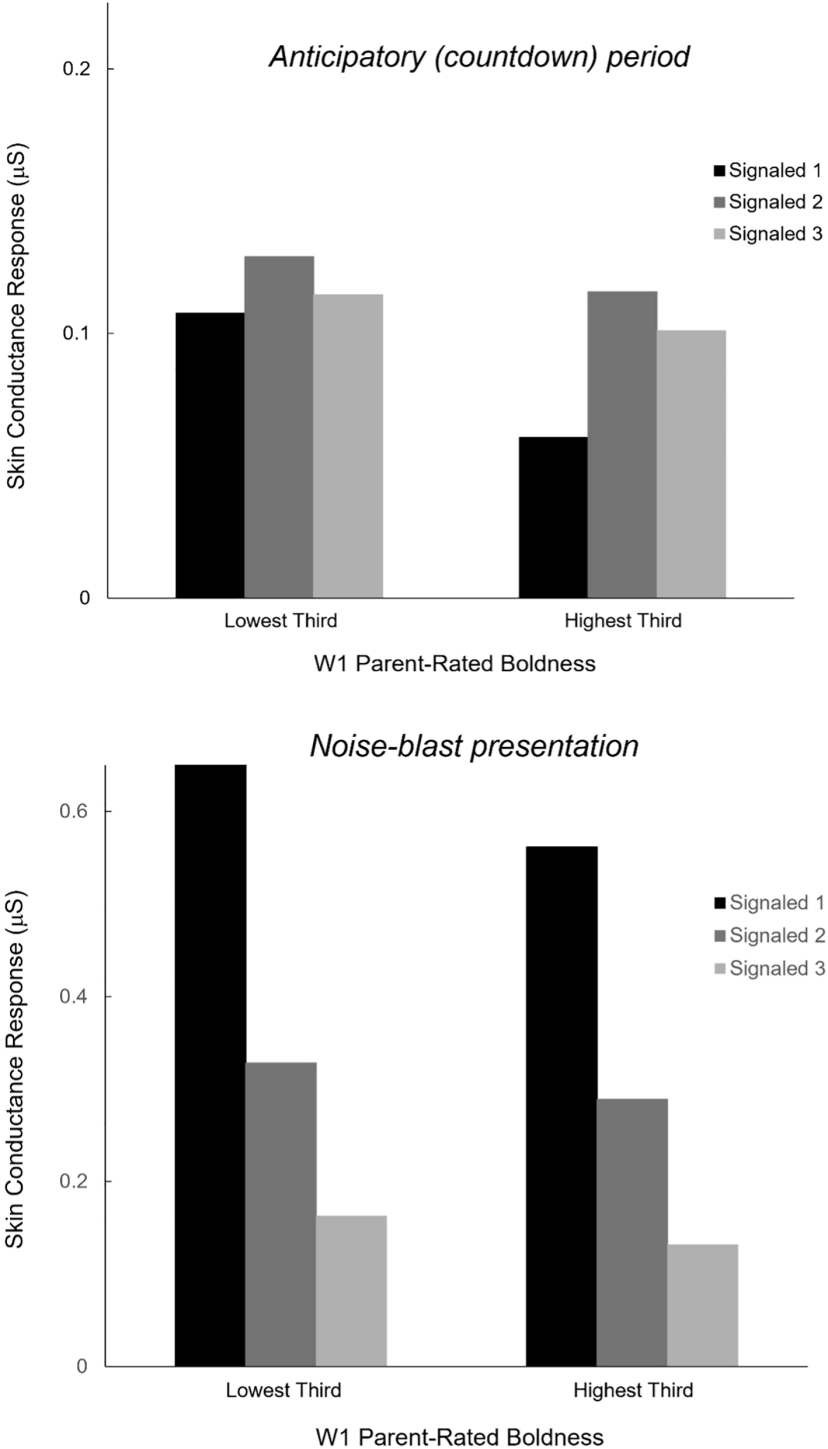
Bar plots depicting mean skin conductance reactivity (SCR), by signaled trial (1-3) of the Wave 1 countdown task, for child participants falling within the lowest versus highest thirds of the distribution of scores on Wave 1 parent-rated boldness. Upper plot: Low- versus high-bold subgroup comparison for SCR during the anticipatory (numeric countdown) period of the three signaled trials. Lower plot: Low- versus high-bold comparison for SCR in response to the noise blast occurring on the three signaled trials.

To illustrate the inverse relations of W1 parent-rated boldness with the two measures of SC reactivity during the W1 countdown task, **Figure 2** depicts mean SCR for the anticipatory periods (upper plot) and noise-blast presentations (lower plot) of the three signaled trials of the task, for participants scoring in the lowest versus highest third of the sample on parent-rated boldness. Of note, W1 anticipatory SCR and noise-elicited SCR were positively correlated with one another (*r* = .42).

### Prospective associations

#### Wave 1 countdown task reactivity with wave 3 parent- and participant-rated triarchic traits

GLMs akin to those for concurrently assessed traits were performed to evaluate whether SC reactivity in the W1 countdown task related to boldness 4–5 years later, as assessed by parents or by participants themselves. Notably, scores for W1 and W3 parent-rated boldness were positively correlated (*r* = .35), indicating temporal stability in parental perceptions of this trait.

The analysis for W1 anticipatory SCR revealed a main-effect association with W3 parent-rated boldness in the same direction as at W1 (i.e., lower anticipatory SC at W1 predicted higher parent-rated boldness at W3), but the association in this case was weaker and nonsignificant, *F*(1, 679) = 1.86, *p* = .17. However, the analysis for SCR to the noise blast revealed a significant main-effect association for W3 parent-rated Boldness, *F*(1, 679) = 4.46, *p* < .04, with children who exhibited smaller SCR to the blast across signaled trials at W1 scoring higher in parent-rated boldness at W3 (*r =* -.08).

In the corresponding analyses focusing on W1 countdown SCR scores and W3 *participant-rated* boldness, main-effect associations of boldness with anticipatory (countdown) SCR and noise-blast SCR were both nonsignificant, *F*s(1, 563) = .10 and 2.23, respectively, *p*s > .13. However, for noise-blast SCR, a significant W3 participant-rated boldness X trial interaction was observed, *F*(2, 1126) = 3.75, *p* < .03, with children who exhibited smaller W1 SC reactivity to the first occurrence of the blast scoring significantly higher in self-rated boldness at W3, *r* = -.10, *p* < .02 (*r*s for SCR to second and third blasts = -.02 and .02, respectively).

The GLMs for W3 parent-rated disinhibition and meanness yielded no main or interactive relations of either trait with SC during the anticipatory or noise-presentation phases of the countdown task, *F*s < 1.17, *p*s > .29, *r*s < |.04|, nor did the GLMs for W3 participant-rated disinhibition, Fs < 1.98, *p*s > .13, *r*s < |.05|. However, a significant predictive association was found for W1 anticipatory SCR with W3 *participant-rated* meanness in the GLM for these measures, *F*(1, 647) = 4.44, *p* < .04, with children who exhibited smaller SCR during anticipation of the signaled noise blasts at W1 scoring higher in self-rated meanness at W3 (*r =* -.08). No such association was found for W3 participant-rated meanness with W1 noise-blast SCR, *F*(1, 647) = 1.25, *p* = .26.

#### Countdown task reactivity and parent-rated boldness at wave 1 as co-predictors of participant-rated antisocial behavior at wave 3

**Table 2** shows bivariate (zero-order) *r*s for the two W1 SC measures and W1 parent-rated boldness with W3 participant-rated ASB scores; **Table 3** shows bivariate *r*s for W3 parent- and child-rated triarchic scale scores with W3 participant-rated ASB scores. In the hierarchical regression analysis for W3 aggressive behavior, anticipatory (countdown period) SC across the three W1 signaled trials did not contribute significantly to prediction when entered at step 1, *F*(1, 626) = 2.15, *p* = .14 (*B* = -.06), whereas W1 parent-rated boldness evidenced incremental prediction (Δ*R*^2^ = .011) at step 2, *F*(1, 625) = 6.81, *p* < .01 (*B* = .10). In the corresponding analysis for participant-rated nonaggressive ASB, on the other hand, the predictive contribution of anticipatory SC at step 1 bordered on significance, *F*(1, 626), = 3.73, *p* = .05 (*B* = -.08), whereas the incremental contribution of W1 parent-rated boldness at step 2 (Δ*R*^2^ = .005) was nonsignificant, *F*(1, 625) = 3.22, *p* = .07 (*B* = .07).

**Table 2:** Bivariate correlations for wave 1 skin conductance (SC) variables and parent-rated triarchic scales with wave 3 participant-rated aggressive and nonaggressive antisocial behavior (ASB).

	W3 Participant-Rated Aggressive ASB	W3 Participant-Rated Nonaggressive ASB
	*r*	*r*
Wave 1 Anticipatory SC	-.06	-.08^†^
Wave 1 Noise-Blast SC	-.04	-.09^*^
Wave 1 Parent-Rated Boldness	.11^**^	.08^†^
Wave 1 Parent-Rated Meanness	.21^**^	.23^**^
Wave 1 Parent-Rated Disinhibition	.25^**^	.23^**^

*N* = 628. ^**^p <.01, ^*^p <.05, ^†^*p* = .05.

**Table 3:** Bivariate correlations for wave 3 triarchic scales with wave 3 participant-rated aggressive and nonaggressive antisocial behavior (ASB).

	W3 parent triarchic scales			W3 child triarchic scales		
	Boldness	Meanness	Disinhibition	Boldness	Meanness	Disinhibition
	*r*	*r*	*r*	*r*	*r*	*r*
W3 Agg ASB	.17^**^	.27^**^	.34^**^	.04	.55^**^	.66^**^
W3 Nonagg ASB	.13^**^	.30^**^	.32^**^	.15^**^	.44^**^	.44^**^

N = 628. Agg, aggressive; Nonagg, nonaggressive. ^**^p <.01.

Turning to the regression analysis for W1 noise-blast SC reactivity and parent-rated boldness as predictors of W3 aggressive behavior, noise-blast SCR did not contribute significantly when entered at step 1, *F*(1, 626) = 1.23, *p* = .27 (*B* = -.04), but parent-rated boldness showed significant incremental prediction (Δ*R*^2^ = .011) at step 2, *F*(1, 625) = 7.06, *p <* .01 (*B* = .11). By contrast, in the analysis for participant-rated nonaggressive ASB, the predictive contribution for noise-elicited SCR at step 1 was significant, *F*(1, 626) = 4.83, *p* = .03 (*B* = -.09), whereas the incremental contribution of parent-rated boldness at step 2 (Δ*R*^2^ = .005) was not, *F*(1, 625) = 3.29, *p* = .07 (*B* = .07). Notably, noise-elicited SCR overlapped minimally with parent-related boldness in predicting nonaggressive ASB, as evidenced by highly similar beta coefficients for each as individual versus joint predictors (*B*s = -.09/-.08 and .08/.07, respectively).^3^

## Discussion

There has been longstanding interest in autonomic reactivity to aversive stimuli as an indicator of fearlessness in psychopathy [e.g., (4, 5, 11, 12, 42)], but most research on this topic has been conducted with adults and further work is needed to determine whether associations observed in older samples, particularly for electrodermal (SC) reactivity, apply to children and adolescents and are attributable more to certain aspects of psychopathy than others. The current study utilized the biobehavioral trait framework of the triarchic psychopathy model and data from the USC RFAB project, a longitudinal twin study that includes measures from different assessment modalities, to test the hypothesis that relations between SC reactivity in an aversive countdown task at age 9–10 would relate to the boldness facet of psychopathy in particular, both concurrently and subsequently (at age 14-15), and would co-predict antisocial behavior at the later age point. We also performed analyses to test more tentative hypotheses for the other triarchic traits – disinhibition and meanness.

### Triarchic traits and electrodermal response during anticipation and to receipt of a noise stressor

#### Concurrent associations

Participants completed the countdown-to-noise task in the first assessment wave of the RFAB project, when they were 9–10 years old. Prior to the first signaled trial of the task, they were told that a loud noise would occur at the end of the countdown period for each of these trials. The pattern of SC reactivity across the three signaled trials in the overall sample was indicative of heightened anticipatory (i.e., countdown-period) activation following receipt of the initial noise blast, and habituation to the stressful impact of the blast itself following its initial occurrence. Specifically, participants as a whole exhibited elevated anticipatory SC during countdowns to the second and third blasts relative to the first, contrasted with elevated SC to the first occurrence of the actual blast relative to the second and third occurrences. The implication is that anxious anticipation generally increased following initial exposure to the noise stressor, whereas familiarity with the noxious noise and enhanced readiness for its occurrence operated to reduce its impact.

In line with our major study hypothesis, the *degree* of SC reactivity that participants exhibited during anticipation of the noise blasts as a whole (i.e., across the three signaled trials) correlated inversely with how bold they were judged to be by their parents at the time of countdown-task completion (i.e., at assessment wave 1). A similar association was found for SCR to the occurrences of the noise blasts (i.e., children exhibiting less reactivity to the blasts across the three signaled trials were rated as higher in boldness by their parents), although this association was somewhat weaker and fell just short of significance. By contrast, W1 anticipatory SCR and noise-blast reactivity were negligibly related to concurrent parental ratings of disinhibition and meanness. Notably, our findings for boldness coincide with results reported by Dindo and Fowles (7) for the closely related trait of PPI fearless dominance, assessed via self-report in a young adult sample. The current study extends this prior work by demonstrating an inverse association between countdown-SCR and parent-rated boldness in a child-aged sample, indicating that children viewed as high in boldness by their parents were less sympathetically aroused during the countdown to the noise blasts and less affected by occurrences of the blasts than children perceived as low in boldness.

Of note, parental ratings of boldness were made using scales developed and validated by Bertoldi et al. (38) and encompassed characteristics of social assurance/assertiveness, lack of anxiety, and behavioral venturesomeness – characteristics that relate in turn to biobehavioral threat sensitivity, a concept that encompasses physiological [e.g., (57)] and task-performance [e.g., (31, 32)] as well as psychological-scale indicators of dispositional fear/fearlessness [for a review, see (58)]. Our finding that W1 countdown SC reactivity covaried with parent-rated boldness indicates that parental perceptions of lower versus higher dispositional threat sensitivity in their children covaried with measurable variations in children’s physiological reactivity within the aversive countdown procedure.

Of further note, a key physiological correlate of dispositional threat sensitivity (57, 59), and for boldness as well (16, 60), is aversive startle potentiation – defined as enhancement of the defensive startle-blink response to sudden acoustic probes presented during exposure to aversive visual images. This physiological index of threat sensitivity was first linked to psychopathy by Patrick et al. (61), who reported a lack of startle potentiation during viewing of aversive pictures (e.g., aimed weapons, attacking figures) in male offenders diagnosed as psychopathic according to Hare’s PCL-R – in particular, those scoring highest on the PCL-R’s affective-interpersonal (Factor 1) features.

Of particular relevance to the current work, a follow-up study by Patrick (62) demonstrated reduced startle potentiation during presentation of a visual display preceding the occurrence of aversive noise blasts among offenders scoring high on PCL-R Factor 1, regardless of their scores on the PCL-R’s impulsive-deviancy (Factor 2) features. This result was interpreted as indicating a lack of defensive motivational priming in relation to the visual cue signaling the upcoming noise blasts. In a report of additional findings from this study, Patrick (63) documented reduced SCR as well to the visual display preceding the noise blasts in relation to higher scores on PCL-R Factor 1, particularly when controlling for scores on PCL-R Factor 2. Given other research identifying boldness as the characteristic that most differentiates PCL-R Factor 1 from Factor 2 (23, 64), this finding for offenders converges with results from the current work with young children and prior research with adult nonoffenders (7) demonstrating reduced aversive-anticipatory SCR in relation to the trait of boldness.

#### Prospective associations

Analyses testing for prospective relations of W1 countdown SC reactivity with triarchic traits assessed at W3 of the RFAB project, when participants were 14–15 years old, also yielded interesting results. Paralleling its concurrent relationship with parent-rated boldness at Wave 1, W1 anticipatory SCR as a whole (i.e., across the three signaled trials) evinced a negative correlation with W3 parent-rated boldness, though this correlation was weaker and did not achieve significance. The negative correlation for W1 *noise-blast* SCR with W3 parent-rated boldness, on the other hand, exceeded its association with W1 parent-rated boldness and was significant. As noted in the Results section, W1 anticipatory SCR and noise-elicited SCR were positively correlated with one another, as were W1 and W3 parent-rated boldness, and supplemental regression analyses indicated that the two SCR measures overlapped in their relations with parent-rated boldness assessed at the two time points (i.e., predictive coefficients for each were reduced when entered together as predictors of W1 or W3 parent-rated boldness). The implication is that W1 anticipatory and noise-elicited SCR were indicative of a common process that related to parental perceptions of children’s boldness across the two assessment waves.

As discussed in the preceding subsection, a viable interpretation based on prior research is that the process in question is one pertaining to distress elicited by the prospect or actual occurrence of an aversive event (i.e., fear). Our study findings suggest that variations in children’s sensitivity to everyday, naturally occurring threats/challenges influence parents’ ratings of boldness-related characteristics. In contrast with these findings for boldness, W3 parental ratings of disinhibition and meanness, like ratings of these traits at W1, correlated negligibly with the two W1 SCR measures – indicating that parental inferences regarding children’s threat sensitivity did not enter into their ratings of these triarchic traits.

Turning to participants’ self-ratings of boldness at W3, one significant finding emerged, entailing an inverse association with a specific aspect of W1 countdown SC – namely, reactivity to the very first of the three signaled noise blasts. This finding is notable in light of the fact that participants as a whole exhibited heightened reactivity to the first versus the second and third blasts, reflected in the main effect of trial for W1 noise-blast SCR. It indicates, in line with prior adult-offender research reporting blunted physiological reactivity to a novel noxious stimulus in relation to the Factor 1 features of PCL-R psychopathy (62, 63), that children who rated themselves as higher in boldness at ages 14–15 were those who were less impacted by the occurrence of a not-previously-experienced stressor. The implication is that participants’ ability to withstand unfamiliar unpleasant events influenced their self-ascriptions of characteristics related to boldness. This interpretation dovetails in turn with prior published work reporting that adults who rate themselves higher in boldness are better able to cope with life stressors than those low in self-rated boldness (65).

No association was observed for either W1 SC reactivity measure with participant-rated disinhibition at W3. However, a significant inverse relationship was observed for W1 anticipatory SCR as a whole with W3 participant-rated meanness. That is, participants who exhibited less anticipatory SC reactivity across the 3 signaled trials of the W1 countdown task rated themselves as higher in meanness at W3. Notably, this prospective relationship for W1 SCR with W3 participant-rated meanness emerged in the absence of any association with parent-rated meanness – either concurrently (at W1) or prospectively (at W3). This pattern of results appears consistent with the triarchic model’s view that dispositional fearlessness plays a secondary role in meanness, with weak affiliative capacity playing a more dominant role (19; see also 25). Lack of anticipatory concern regarding stressors such as potential punishments and social disapproval may connect more to children’s self-perceptions of meanness-related characteristics such as toughness, superiority, and aggressive competitiveness, whereas weak affiliation may relate more to parental perceptions of meanness-related characteristics such as low empathy, antagonism, and exploitativeness.

### Early electrodermal stress response and parent-rated boldness as prospective predictors of later antisocial behavior

Given the above-noted relationships between W1 countdown SCR and W1 parent-rated boldness, a question of interest was whether these two indices of dispositional threat sensitivity – one physiological (SCR) and the other psychological (boldness) – would overlap in terms of their predictive relations with later-assessed clinical outcomes of interest (i.e., aggressive and nonaggressive ASB at W3) or operate as separate prospective predictors of these outcomes. Hierarchical regression analyses performed to address this question revealed that boldness as rated by parents at W1 operated as a unique predictor of child-reported aggressive ASB at W3: When entered as an additional predictor following either W1 anticipatory or noise-elicited SCR, neither of which contributed on its own to prediction of this outcome, boldness evidenced significant incremental prediction. By contrast, W1 parent-rated boldness did not contribute significantly to prediction of W3 nonaggressive ASB over/above W1 anticipatory or noise-elicited SCR, which evidenced near-significant (*p* = .05) and significant relations, respectively, as individual predictors. Comparison of the beta coefficients for parent-rated boldness as a joint versus individual predictor indicated that its relationship with nonaggressive ASB, which was somewhat weaker than for noise-elicited SCR, was largely independent of SCR’s predictive association.

The finding that W1 parent-rated boldness was uniquely predictive of W3 aggressive ASB whereas W1 countdown-SCR was more strongly predictive of W3 nonaggressive ASB indicates that these alternative-modality measures, though covarying to some extent, also tapped distinct aspects of fearlessness (low threat sensitivity; 24) that influenced behavior in different ways across time. Notably, the trait of boldness encompasses facets of social dominance and venturesomeness that are visible in overt behavior, along with low anxiousness, an internal-experiential characteristic that is less readily observable (66, 67). It seems likely in particular that tendencies toward dominance reflected in parental ratings of boldness would have been predictive of later aggressive ASB, given documented links between social dominance and aggressiveness (e.g., 68, 69). On the other hand, lack of anxiousness (alternatively known as “stress immunity” [14] versus “potential threat” sensitivity [70]) may comprise the element of fearlessness tapped by reduced countdown SC reactivity that was more predictive of nonaggressive ASB. Individuals lacking in anticipatory anxiety would be less likely to consider the adverse consequences of actions aimed at achieving immediate gratification. Of note, this perspective accords with Fowles’ (71) classic view of a deficit in the brain’s punishment-processing (behavioral inhibition) system as the basis of reduced aversive-anticipatory SC reactivity in psychopathy [see also (72)].

### Study limitations, implications, and future directions

Given its longitudinal design, comparatively large sample, and triarchic conceptual focus, the current work represents a novel and important addition to the existing literature on autonomic reactivity to stress as a factor in psychopathy and ASB. However, some notable study limitations warrant mention. One is that electrodermal reactivity, which reflects sympathetic nervous system activation, is indicative of autonomic arousal broadly rather than of defensive (fear) reactivity specifically (61, 73). It would have been beneficial if data for startle-blink potentiation had been collected in the countdown task, as a more specific index of fear activation (74). This would have enabled us to corroborate prior adult studies demonstrating reduced startle potentiation during aversive cueing in offenders scoring high in affective-interpersonal features of psychopathy (62) and community participants high in boldness (16, 60). Another study limitation is that the clinical outcomes of interest at W3, aggressive and nonaggressive ASB, were assessed exclusively via participant self-report; it would have been beneficial to have some other source of information regarding ASB (e.g., school disciplinary records), given limitations of self-report data. However, mitigating this concern somewhat, the W1 measures that we examined as predictors of W3 ASB were from separate measurement modalities – i.e., parent ratings (of triarchic traits) and physiological response (from the countdown task). The relations observed for these W1 predictors with W3 ASB attest to the validity of participant-report scores for the latter. A further limitation is that the participant sample, because it was selected to be representative of the urban population from which it was drawn (i.e., the greater Los Angeles area), was nonclinical in nature and not enriched for psychopathology. Some use of preselection for elevated risk (e.g., parental psychopathy scores; family history of ASB, or externalizing problems more broadly) would have resulted in greater representation of high psychopathy/ASB participants in the sample as a whole and potentially larger effect sizes for predictive associations.

As a further point, it must be acknowledged that the significant associations between countdown SCR and boldness scores in the current study, though predicted on the basis of prior research, were small in magnitude (i.e., -.07 to -.10). Factors contributing to this low magnitude of association likely included the difference in measurement methods (i.e., physiology versus parent-/participant-report), construct dissimilarity (i.e., lab-stressor reactivity versus dispositional threat sensitivity), and the young age of participants (ages 9-10) at the time of the countdown-task assessment. Measurement-method dissimilarity has been discussed in particular as a key constraint on the magnitude of observed effects in research aimed at identifying physiological correlates of clinical-psychological conditions more broadly (21). A potential solution to this problem may lie in operationalizing clinically-relevant traits such as threat sensitivity through combined use of physiological and report-based measures (42, 75). The current study is valuable in this regard, as it directly corroborates prior work by Dindo and Fowles (7) and thereby points to reduced countdown-SCR as a physiological indicator of threat sensitivity that could be incorporated into a multi-method assessment of this biobehavioral trait [see (57), for initial work along this line].

Notwithstanding these limitations, findings from the current longitudinal work extend prior cross-sectional research and have important implications for our understanding of psychopathy and its facets, and their relations with different forms of ASB. Corroborating prior research, our findings indicate that reduced electrodermal reactivity in the classic countdown task (11) relates specifically to the boldness facet of psychopathy, which comprises part of the affective-interpersonal component of psychopathy (6, 23). Notably, while overall psychopathy as indexed by the PCL-R is uncorrelated with anxiety (8), its interpersonal features – which connect most robustly to boldness (23) – relate negatively to anxious traits (i.e., stress reactivity, distress, neuroticism) as well as to fearfulness (76). Extending prior research, our findings demonstrate that reduced countdown-SC reactivity at age 9–10 not only covaried concurrently with parental ratings of boldness, but also prospectively predicted parent-rated boldness along with participant-reported engagement in nonaggressive ASB, five years later. The implication is that countdown-SC reactivity taps a stable aspect of emotional (in)sensitivity that relates both to the persistence of observable manifestations of boldness across time, and to risk for engagement in antisocial acts as individuals progress from childhood into adolescence.

Another notable finding was that countdown-SCR and parent-rated boldness prospectively predicted different forms of ASB – i.e., nonaggressive versus aggressive variants. An important implication of this finding is that physiological and report-based measures of dispositional characteristics relevant to psychopathology – in the present case, low threat sensitivity as an attribute related to psychopathic personality – can be expected to covary only modestly (77) and may complement one another as predictors of clinical outcomes owing to their nonshared variance. From this perspective, a multi-method approach to operationalizing clinically relevant individual-difference characteristics may provide unique advantages for identifying early risk for psychopathology and predicting distinct trajectories and outcomes of dispositional risk (75). An important priority for future research will be to identify other physiological correlates of the distinct dispositional facets of psychopathy – boldness, meanness, and disinhibition – and evaluate their overlapping versus unique predictive validity with respect to clinically meaningful criterion variables assessed in different ways (42).

Another key implication of the current work relates to the concept of boldness as a normative bipolar trait dimension (19, 24), substantially related (in reverse) to the bipolar dimension of dispositional threat sensitivity (59, 78), that confers risk for different clinical outcomes at its high versus low extremes. Specifically, as a facet of psychopathy, high boldness relates to maladaptive propensities including narcissism, risk taking, and (as shown in the current study, and other published work – e.g., 48, 79) antisocial behavior. By contrast, low boldness is associated with heightened susceptibility to anxious-depressive (internalizing) problems, in particular focal fear (phobic) conditions (80). Likewise, dispositional threat sensitivity – defined as the latent dimension underlying various self-report measures of fear and fearlessness (59) – relates positively to fear-disorder symptomatology, both phenotypically (81) and genotypically (82, 83). An important direction for future research will be to further examine the extent to which common etiological influences contribute to psychopathy on one hand and fear-related disorders on the other. Of particular interest will be studies examining whether physiological indicators such as countdown-SC reactivity and aversive startle potentiation co-predict outcomes of these differing types, and research evaluating whether the presence versus absence of particular gene combinations confers risk in one direction versus the other.

In conclusion, the current work importantly extends prior research documenting diminished stress reactivity, in the form of reduced electrodermal response within an aversive countdown task, in high-psychopathic individuals. Our findings point to boldness, the facet of psychopathy theorized to relate most to dispositional fearlessness (19), as the basis for this association – and provide evidence that diminished countdown-SC reactivity in childhood is associated with persistence of fearless-dominant (bold) propensities across time and associated adverse outcomes in the form of ASB.

## Author’s notes

The content of this article is solely the responsibility of the authors and does not necessarily represent the official views of the U.S. government, Department of Defense, Department of the Army, Department of Veterans Affairs, or U.S. Recruiting Command.

## Data Availability

Data are obtainable in de-identified form upon request from Laura Baker, lbaker@usc.edu.

## Footnotes

1 Wave 3 was chosen as the follow-up assessment point because this was the first point at which items comprising the triarchic trait scales used in the current study (see Method section) were available for both child participants and their parents. Parent ratings of the triarchic traits were expected to be less valid at subsequent waves, when participants were less observable due to spending less time at home.

2 The items comprising each of the RFAB-Tri scales, and the inventories from which they were drawn, are provided in the online Supplement to the article by Bertoldi et al. (38) reporting on the development and initial validation of these scales. The web address for the Supplement to this article is: https://doi.org/10.1017/S0954579420002060

3 We did not perform counterpart hierarchical regression analyses to test for incremental prediction of ASB outcomes from W1 parent-rated disinhibition or meanness because these triarchic traits, unlike boldness, did not covary with either of the step 1 SCR predictors. However, of note, W1 parent ratings for each of these traits did correlate significantly (*p*s < .001) with both aggressive and nonaggressive ASB at W3: *r*s for W1 meanness = .20 and .23, respectively; rs for W1 disinhibition = .24 and .22. Additionally, W1 parent-rated disinhibition and meanness predicted these W3 ASB outcomes independently of one another, as evidenced by significant regression *B*s (*p*s < .002) for each as co-predictors: *B*s for aggressive ASB = .19 and .13, respectively; *B*s for nonaggressive ASB = .15 and .17. Also notable was that W1 parent-rated boldness contributed incrementally over W1 parent-rated disinhibition and meanness to prediction of each of these W3 ASB outcomes: regression *B*s for aggressive and nonaggressive ASB = .12 and .08, respectively, *p*s < .03.
